# Transient chromatin properties revealed by polymer models and stochastic simulations constructed from Chromosomal Capture data

**DOI:** 10.1371/journal.pcbi.1005469

**Published:** 2017-04-03

**Authors:** Ofir Shukron, David Holcman

**Affiliations:** 1 Institute of Biology, Ecole Normale Supérieure, Paris, France; 2 Mathematical Institute, University of Oxford, Oxford, United Kingdom; CNAG - Centre Nacional d’Anàlisi Genòmica and CRG - Centre de Regulació Genòmica, SPAIN

## Abstract

Chromatin organization can be probed by Chromosomal Capture (5C) data, from which the encounter probability (EP) between genomic sites is presented in a large matrix. This matrix is averaged over a large cell population, revealing diagonal blocks called Topological Associating Domains (TADs) that represent a sub-chromatin organization. To study the relation between chromatin organization and gene regulation, we introduce a computational procedure to construct a bead-spring polymer model based on the EP matrix. The model permits exploring transient properties constrained by the statistics of the 5C data. To construct the polymer model, we proceed in two steps: first, we introduce a minimal number of random connectors inside restricted regions to account for diagonal blocks. Second, we account for long-range frequent specific genomic interactions. Using the constructed polymer, we compute the first encounter time distribution and the conditional probability of three key genomic sites. By simulating single particle trajectories of loci located on the constructed polymers from 5C data, we found a large variability of the anomalous exponent, used to interpret live cell imaging trajectories. The present polymer construction provides a generic tool to study steady-state and transient properties of chromatin constrained by some physical properties embedded in 5C data.

## Author summary

Chromatin organization remains poorly understood and polymer models are used to reconstruct such organization, to reveal hidden structures and to quantify genomic interactions. We use a generalized Rouse model (a linear chain of beads connected by springs) with additional interacting molecules that allow stable loop formation. The polymer models are constructed using the minimal number of binding molecules, positioned according to the encounter probability matrix obtained from experimental chromosomal capture data. We determine the conditional encounter probability of 3 key loci regulating gene inactivation from our calibrated polymer model. Using polymer simulations, we generate single particle trajectories and explore their transient properties. The present results suggest that the heterogeneity of anomalous exponents measured in live cell imaging is due to the large combinatorics in reconstructing the chromatin organization from Chromosomal Capture data. The present method and algorithms are generic and can be used to reconstruct a polymer model at a given scale from any Chromosomal Capture data.

## Introduction

Chromatin is organized in heterogeneous sub-regions of various sizes, as recently revealed by Chromosome Capture (5C) data [1, 2]. This multiscale organization is generated by short and long-range genomic interactions between DNA segments, observed in the statistics of a large number of cells. Mammalian chromatin at a resolution of 3kb, [3, 4] contains an organization at 1Mbp scale, where several sub-structures are enriched with intra-connectivity, reflecting an increased encounter probability (EP) between genomic segments. This increased EP is described in the two-dimensional encounter frequency (EF) matrix, containing diagonal blocks called Topologically Associating Domains (TADs) [3, 5]. TADs are associated with gene regulation [3], DNA replication timing [6], DNA entanglement or cross-linking by molecules such as cohesin, CTCF [7] and condensin [3]. Cross-linking between chromatin sites are precisely the events sampled by Chromosome Capture data (3C, 4C, 5C, HiC) [3, 8], and single cell HiC confirms that positions of cross-links can vary between cell types and phases [9]. In that context, TADs represent average chromatin conformations, characterized by a higher numbers of binding molecules compared to non-TAD regions.

Over the past ten years, polymer models have been used to analyze statistics hidden in Chromosome Capture (CC) data and to characterize the decay of the encounter probability with the genomic distance *s*. For example, the EP between two monomers *A*, *B* for a (linear) Rouse polymer decays with *s*^−3/2^, thus the exponent is 3/2 [10]. A range of decay exponents lower than 3/2 can be produced by other polymer models, where stable transient loops are formed between segments [11]. By varying the number of loops, a large range of chromatin configurations can be generated and the associated polymer characteristics are reflected in the EP decay exponent. By including transcriptional information, dynamic-loop model [11, 12] can reproduce chromatin looping associated with transcriptional activity. Similar polymer models describe chromosomal territories [13], suggesting that inactive genes are located inside these territories. The strings-and-binders-switch polymer model consists of reversible binding between specific genomic segments located in close proximity, when an additional diffusing molecule is present at the binding site. In an extension of this model, genomic segments having similar epigenomic state can directly interact [14], revealing the phase diagram of polymer configurations. These models are used to interpret the contact probability decay in the Hi-C data [15–18]. In a new class of polymer model, each monomer interacts with any other through a potential well [5, 19], where pairwise interaction between monomers is represented by either an attractive or repulsive potential. The parameters of the model are extracted from data by minimizing the chi-square norm between the EP empirical and Monte-Carlo simulation matrices. However, this highly connected model does not translate easily into molecular binding because the nature of these potential wells does not have a direct physical interpretation. When this model is applied to the region containing the X inactivation center of mammalian embryonic stem cells, it predicted novel ensemble of polymer configurations, representing TAD structures present in the 5C data. Other minimization procedures were used to inter-chromosomal distances at a large-scale resolution of 1Mbp [20].

Polymer models have also been used to interpret single particle trajectories (SPTs) of tagged DNA locus [21–27] revealing that chromatin is constantly remodeled. SPTs are characterized by their anomalous behavior, which can deviate significantly from classical diffusion and are usually quantified by the mean-square displacement (MSD). In that context, a model with a minimal number of parameters is still needed to account for both types of data: 1) the EPs decay rate and 2) dynamical parameters such as the anomalous exponent extracted from SPTs. In the absence of a systematic procedures to convert the EP into a polymer model with a similar EP decay rate as the one presented in the 5C data, the connection between 1) and 2) was left open.

We present here a general computational and algorithmic procedure to estimate parameters of a randomly cross-linked polymer model following the 5C protocol. The procedure consists in constructing an ensemble of polymer models from the EP of 5C by randomly cross-linking monomers and in resolving the difficulty of assigning the minimal number of sparse interactions between monomer pairs. These interactions can be directly interpreted as binding molecules. The construction of the polymer model starts with the Rouse model [10], which consists of beads linearly connected by harmonic spring. We started with the coarse-grained Rouse polymer that describes accurately the statistics of the chromatin below a scale of few Mbp [28, 29]. To further constrain monomer interactions, we determine monomer connectivity from the 5C data of mammalian X chromosomes. The construction procedure is divided into two steps: first, to account for heterogeneity in the 5C data, we added a minimal required number of connectors (cross-links) between genomic sites chosen at random that can reproduce TAD blocks. We show that the number of random connectors to be added is uniquely determined from data. However, this step is insufficient to recover the EP decay peaks contained in the 5C data. Thus, in the second step, we account for consistent long-range interaction present in the EP matrix within and between TADs. We calibrate our model by requiring that the EP matrix, constructed from simulations of the polymer, has the same decay exponent (for each monomer) as that of the empirical data. These calibrated polymer models allow us to study transient properties and to estimate the conditional encounter probability and the first encounter time between three specific genomic sites.

Although the generalized Gaussian polymer model we are using here is well known, our reconstruction using minimal number of short and long-range connectors derived from empirical EP is new and permits generating novel statistics describing transient gene regulation. By exploring the statistics of simulated SPTs (by computing the anomalous exponent [21–24]), we further show that the large heterogeneity of the anomalous exponent present in live cell imaging can be explained by random binding locations on the chromatin that can vary from cell to cell.

## Results

### The encounter probability of coarse-grained 5C data

**Fig 1:**
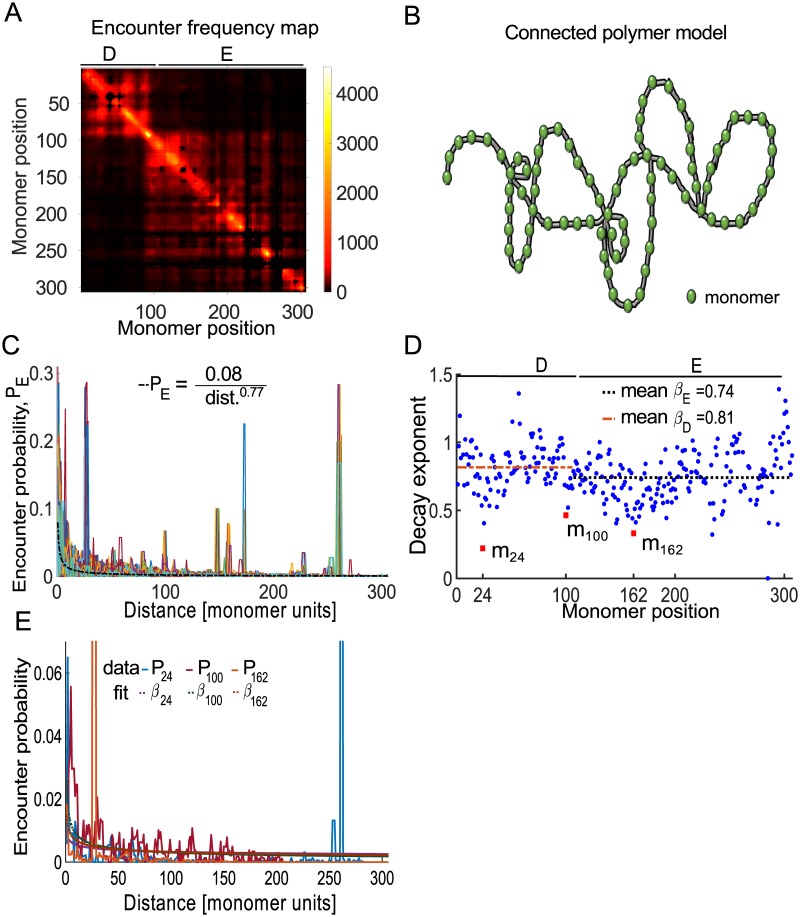
Statistics of conformation capture data. **A**. Average encounter frequency map of two 5C replica spanning ≈1Mbp genomic region containing two Topologically Associating Domain (TAD) D (monomers 1–106) and TAD E (monomers 107–307) [3], where the map was coarse-grained into 307 monomers of size 3kbp [5]. **B**. Schematic representation of a polymer model with randomly connected monomers. **C**. Empirical encounter probability *P*_*n*_, for monomer *n* plotted with respect to the genomic distance *d* [monomer units], reveals long-range interactions (localized peaks). *P*_*n*_ are fitted with functions *Ad*^−*β*^, where *β* is the decay exponent and *A* the normalization factor. For the mean encounter probability $\bar{P}$, the value of the parameters are *A* = 0.08 and *β* = 0.77 (thick red curve). **D**. Distribution of the *β*_*n*_ exponents (*n* = 1‥307) (blue dots): Monomers *m*_24_, *m*_100_, *m*_162_ (red square dots) with *β*_24_ = 0.22, *β*_100_ = 0.46, and *β*_162_ = 0.33, respectively, accounts for high peaks (first and last), while the middle one corresponds to the boundary between TADs. **E**. Encounter probability *P*_*n*_ for monomers n = 24, 100, and 162, corresponding to local minima shown in box D.

We previously described how we construct a polymer model from a symmetrized 5C matrix *M* (Fig 1A). By symmetrizing the EP matrix, we averaged-out asymmetrical fluctuations. The 5C data we used represent a sub-region of the X chromosome (≈ 92*kbp*), that was previously segmented into two regions called Topological Associating Domains (TADs) D and E [3]. The matrix *M* was further coarse-grained by binning the encounter frequencies into 307 monomers of 3*kbp* [5], where TAD D (resp. TAD E) is represented by the first 106 monomers (resp. 107–307), as shown in Fig 1A. We introduce a general polymer model (Fig 1B) with arbitrary configuration, the properties of which will be extracted from empirical 5C matrix *M*.

The encounter probabilities between monomer *m* and monomer *n* are computed from the experimental 5C matrix *M* (see Material and Methods) by

$$\begin{array}{c} P_{e}(|m-n||n)=\frac{M_{n,n+|m-n|}+M_{n,n-|m-n|}}{\sum_{m=1}^{N}M_{n,m}}, \end{array}$$ (1)

which depends on the genomic distance |*m* − *n*| (Fig 1C). Although the average encounter probabilities decays with |*m* − *n*| for each *n*, they contain peaks that reflect consistent long-range interactions between monomers. To quantify the decay of the EP, we fitted its average value $P_{e}(|m-n|)=\frac{1}{N}\sum_{n=1}^{N}P_{e}(|m-n||n)$ (black dotted line in Fig 1C) with the function

$$\begin{array}{c} \tilde{P}(|m-n|)=\frac{C}{{\left|m-n\right|}^{\beta }}, \end{array}$$ (2)

where *C* and *β* > 0 are two constants. For a Rouse polymer, the EP function $\tilde{P}$ is characterized by a decay exponent *β* = 3/2 [10]. Fitting Eq 2 to data, revealed that *β* = 0.77, from which we concluded that the polymer model should be modified to account for higher compaction than allowed by a Rouse polymer [30].

To better account for the heterogeneity in the EP of each monomer, we plotted the distributions of the exponent *β*_*n*_ for *n* = 1‥307 along the polymer (Fig 1D blue dots). The exponents *β*_*n*_ were extracted by fitting the function 2 to the empirical EPs Eq 1. The large variability in *β*_*n*_, *n* = 1‥307 reflects the local heterogeneity of the chromatin architecture at the current scale (a monomer represents 3kbp). The average value *β* for TAD D and E was found to be *β*_*D*_ = 0.81 and *β*_*E*_ = 0.74 respectively. The local minima of *β* deviated significantly from the mean values (Fig 1D red squares), where the deviation is 2–3 times the standard deviation computed from the *β* values in each TAD. The minima may represent chromatin features (Fig 1E) such as specific long-range interactions or boundary between chromatin sub-domains. Indeed, point *m*_100_ (around monomers 102–107 in Fig 1D red) is located at the boundary between TAD D and E, while *m*_24_ and *m*_162_ are characterized by strong long-range interactions (Fig 1E).

To conclude, the distribution of *β* values extracted from the EP is quite heterogeneous, which can disclose chromatin subregions and long-range strong interactions. We shall account in the next two sections for these characteristic features and include in the polymer model both random and persistent long-range connections between monomers.

### Encounter probability in the random loop polymer model

**Fig 2:**
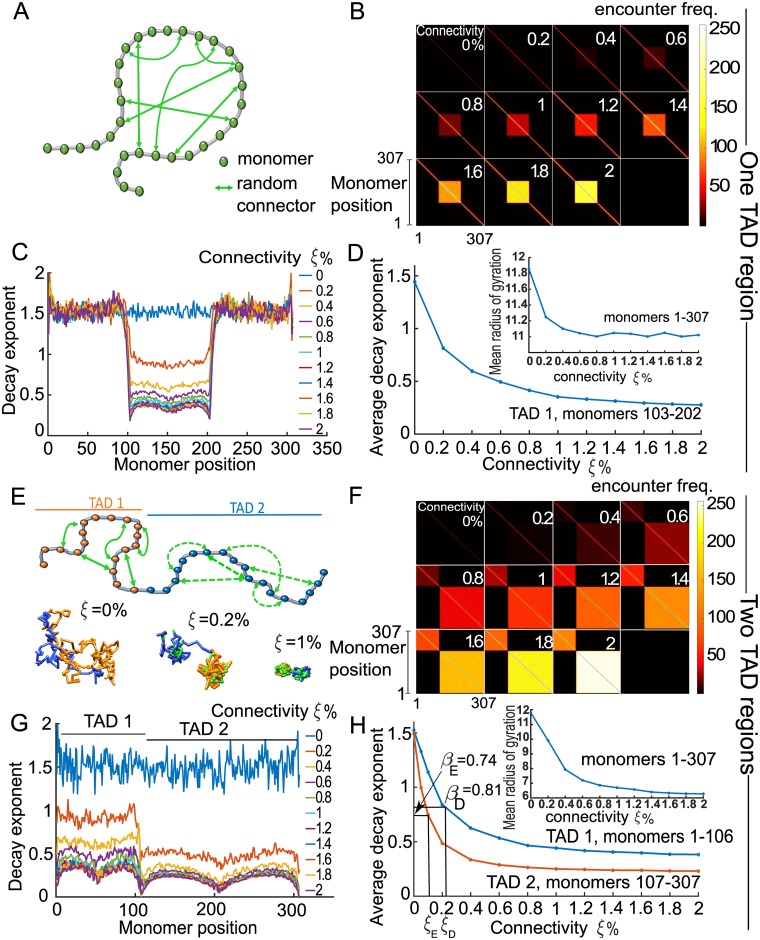
Statistics of simulated generalized Rouse polymer chain for various connectivity in one and two sub-regions. **A**. Schematic bead-spring chain connected at random positions (two-sided green arrows) between non-nearest-neighbor monomers. **B**. Encounter frequency maps of a 307 monomers chain. Connectors are added randomly between monomers 103–202 for each realization. The connectivity *ξ* (number of connectors) increases from 0 to 2%. **C**. Distribution of *β*_*n*_ (*n* = 1‥307) fitted by the function *Ad*^−*β*^, where *d* is the distance along the chain [monomer units], to the encounter probabilities of numerical simulation. **D**. Average value of *β* for monomers in the interval 103–202 with respect to the connectivity percentage *ξ*. **E**. Schematic polymer chain, where two defined regions: monomers 1–106 (TAD 1, orange circles) and monomers 107–307 (TAD 2, blue circles), are randomly connected (green arrows). No connections were added between the two TAD regions. Lower panel: three snapshot realizations of a random loop chain with TAD 1 (orange) and TAD 2 (blue) and random connectors (green) for three increasing values of connectivity *ξ* = 0, 0.2, 1%. **F**. Encounter frequency maps showing two TAD regions for an increasing number of random connectors. **G**. Distribution of *β*− exponent for *ξ* ∈ [0, 2]%, showing the border (n = 106) effect between TADs. **H**. Average *β* over each TAD 1 (blue) and TAD 2 (orange) for *xi* ∈ [0, 2]. The curves decrease until plateau at 0.42 (0.24) for TAD 1 (resp. TAD 2). We use these curves to recover the connectivity percentage *ξ* of the experimental TAD D, with *β*_*D*_ = 0.74 (resp. TAD E with *β*_*E*_ = 0.81) for which *ξ*_*D*_ = 0.23% (resp. *ξ*_*E*_ = 0.12%.).

To determine the level of connectivity of the generalized Rouse polymer which reproduce the EP-decay using a prescribed exponent *β*, we first studied the case of one TAD-like region using the simulation of a 307 monomer chain. For each realization, we added connectors between random non-nearest neighbor monomer-pairs in the subregion 103–203 (Fig 2A). The number of connectors, or the connectivity percentage *ξ* (fraction of the number of connected monomer-pairs to the maximum, described in Materials and Methods), was increased in the range 0–2%. By adding connectors, the EP between distant monomers has increased, as presented in the EP-matrix (Fig 2B). In contrast, outside the region 103–203 the EPs were similar to the case *ξ* = 0 (linear chain), showing that the connected region did not affect the EP in the non-connected ones. At this stage, we have shown that adding random connectors allows recovering the shape of TAD regions.

To find the minimal numbers of connectors necessary to recover a given TAD, we aim of elucidating the relationship between the connectivity percentage *ξ* and the decay exponent *β*. For that purpose, we simulated an ensemble of polymers to their relaxation time (Materials and Methods), and used the equilibrium configuration to estimate the EP of each monomer for *ξ* ∈ [0, 2]%. We calculated the exponent *β* by fitting the function 2 to the simulated encounter probability data: the values of *β*_*n*_ for *n* ∈ [103, 203] decreased with *ξ*. Indeed, for *ξ* ≈ 0.2%, the coefficients *β*_*n*_ decreases below the Rouse exponent (equals to *β*_*Rouse*_ = 1.5), indicating compact polymer configurations. For *ξ* = 2%, the mean decay exponent *β*_*n*_ and *n* = 103–203 was $\bar{\beta }=0.47$, with a minimal value 0.42 obtained for the boundary monomers 103 and 203 (Fig 2C). These results confirm that polymer condenses when *ξ* increases, as confirmed by computing the mean radius of gyration *R*_*g*_ [10], showing a decay from *R*_*g*_ = 11.9 for *ξ* = 0 to *R*_*g*_ = 11 at *ξ* = 2% (measured for 307 monomers, Fig 2D). Values of *β* outside the TAD region (monomers 1 to 102 and 204–307) were mostly unchanged, fluctuating around *β* = 1.5, confirming that statistical properties of a Rouse chain are unaltered when connectors are added to the middle region (*n* = 103 – 203). Finally, the average value of *β*_*n*_ (computed over *n* = 103 – 203) versus *ξ* is shown in Fig 2D and, as we shall see, will serve to extract the connectivity percentage *ξ* from the empirical data.

To reproduce the two TADs D and E of the X-chromosome, we started with a polymer of 307 monomers and added connectors randomly (green arrows) between monomers 1–106 and between monomers 107–307, as described in Fig 2E upper panel). This partition follows the empirical TAD segmentation described in [3, 5] at the scale 3kbp of the polymer model. Three polymer realizations for *ξ* = 0, 0.2, 1% are shown in Fig 2E bottom panel, showing polymer condensation into two distinct regions. The EF matrix shows that two TAD-like regions, named TAD1 and TAD2 (Fig 2F), emerge as the connectivity *ξ* increases from 0 to 2%. To extract the exponent *β* (Fig 2G, colored curves) we fitted the function 2 to the EP matrix for each monomer inside TAD1 and 2. In both cases, the exponent *β* decreased below *β*_*Rouse*_ = 1.5 (*ξ* = 0% blue curve) and for *ξ* = 0.2%, 11 and 39 random connectors were added for TAD1 and TAD2, respectively. The boundary between TADs is characterized by an abrupt decay of the *β* value, reflecting high long and short-range encounters. The average *β* exponent (averaged over each TAD), plotted with respect to the connectivity *ξ* (Fig 2H), was used to determine the number of connectors necessary to reconstruct the empirical data. Indeed, we extracted from the EP matrix (Fig 1A) that *β*_*D*_ = 0.81, *β*_*E*_ = 0.78 the associated connectivity percentages *ξ*_*D*_ = 0.12, *ξ*_*E*_ = 0.23, respectively (Fig 2H). To conclude, we obtain the minimal number of random connectors to be added on a generalized Rouse polymer such that the decay exponents of the reconstructed and empirical EP-matrix are as close as possible.

### Incorporating long-range empirical interactions in the polymer model

**Fig 3:**
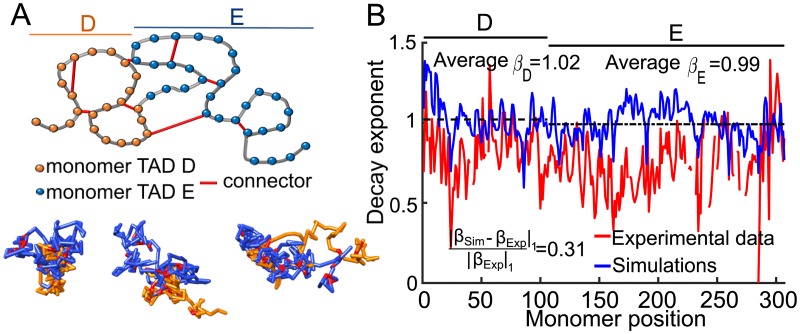
Effect of persistent long-range connectors on polymer folding. **A**. Upper panel: schematic representation of the bead-spring polymer model with added fixed connectors (red) representing specific long-range monomer interactions (peaks) shown in Fig 1C. Lower panel: three different realizations of the same polymer, showing TAD D (orange), TAD E (blue), and fixed connectors (red). **B**. Simulated (blue) and experimental (red) *β* exponent of the fitted encounter probability. The polymer model contains only specific long-range interactions. Average *β* values for TAD D and E are *β*_*D*_ = 1.02 (resp. *β*_*E*_ = 0.99).

A key feature present in the 5C EF-matrix (Fig 1A) is the ensemble of persistent long-range interactions between monomers (Fig 1C). To account for these interactions, we connected monomers corresponding to off-diagonal local maxima of the EF matrix for which their EP exceeds that of nearest neighboring monomers threshold (Materials and Methods). We found 24 long-range connections: 7 (resp. 13) within TAD D (resp. E) and 4 across the two (see SI for the list of monomers pairs) as shown in Fig 3A. We adjusted the spring constant *κ*_*m*, *n*_ between monomer *m* and *n* corresponding to consistent long-range interactions, based on the values of their EPs (Materials and Methods). The scaled coefficient *κ*_*m*, *n*_ are summarized in the SI, and were found to be 1.1 to 3 times higher than the spring constant assigned to connectors of the linear backbone (*κ* = 0.97). As revealed by simulations of a Rouse polymer with fixed long-range connectors, the polymer configuration are condensed, characterized by a radius of gyration of about *R*_*g*_ = 9.1 (compared to *R*_*g*_ = 12 for the Rouse chain). Three realizations of the polymer are shown in Fig 3A.

To quantify the effect of adding consistent long-range connections on the EP decay, we simulated a Rouse chain containing 307 monomers with the addition of fixed monomer connectivity extracted from the peaks of the empirical EP-matrix. We computed the decay exponents *β*_*n*_ of each monomer by fitting the function 2 to the EPs from simulations, and compared it to the ones computed from the experimental data (Fig 3B). To estimate the quality of the reconstruction we use the *L*_1_-norm, defined for a function *f* by |*f*|_1_ = ∑_*k*_|*f*(*k*)|, and computed the difference between the experimental *β*_*Exp*_ and simulated *β*_*Sim*_ curves normalized by the norm ∣*β*_*Exp*_∥_1_ and we find

$$\begin{array}{c} \frac{∥\beta_{Exp}-\beta_{Sim}{∥}_{1}}{|\beta_{Exp}{∥}_{1}}=0.312. \end{array}$$ (3)

The experimental values *β*_*n*_ (Fig 3B red) were generally lower than the ones obtained from simulations (blue), indicating that the reconstructed chromatin polymer is less condensed for both TADs. The mean *β* values for TAD D and E were quite similar with *β*_*D*_ = 1.02 and *β*_*E*_ = 0.99. Therefore, we conclude that long-range connectors are insufficient to reproduce the statistics of the 5C data.

### Combination of random loops and long-range interactions to construct a polymer model of a TAD

**Fig 4:**
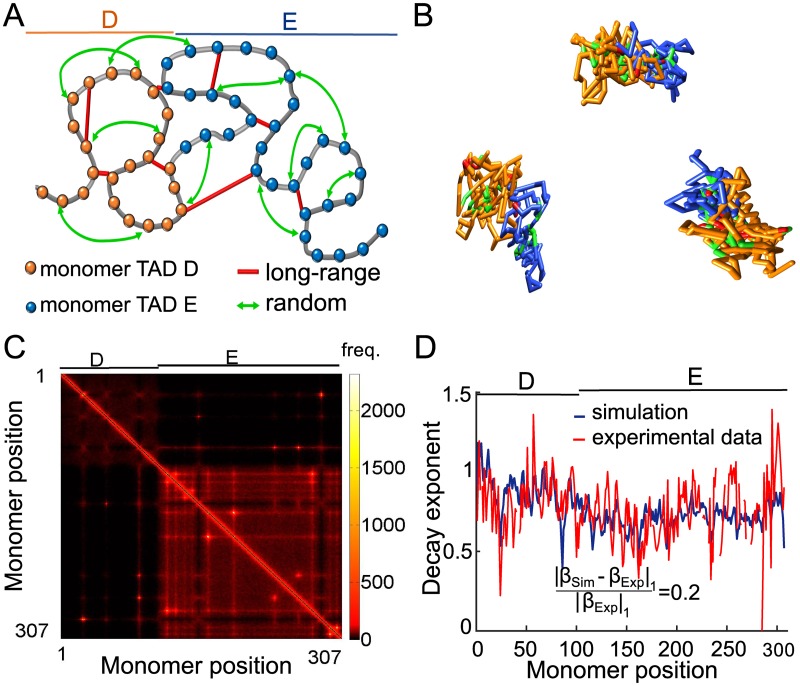
Coarse-grained reconstruction of chromatin using extracted random loops and connectors corresponding to peaks of the 5C data. **A**. Schematic polymer model, where TAD D (orange, monomers 1–106), and TAD E (blue, monomers 107–307) are recovered by random loops (green arrows) using the connectivity *ξ* and persistent long-range connectors (red bars). **B**. Three realizations of the polymer model. **C**. Encounter frequency matrix of the simulated polymer model, showing two TADs where off-diagonal points indicate fixed connectors. **D**. Comparison between *β* computed from experiments and simulations data.

We previously evaluated separately the effect of adding random connectors and fixed long-range interactions on the EPs. We computed the decay exponent *β* and compared it with coarse-grained 5C data. We now combine these two constraints, such that specific and non-specific connectors are added to each realization of a generalized Rouse polymer (Fig 4). We first find the connectivity percentage matching that of the experimental data in each TAD *ξ*_*D*_ = 0.23% and *ξ*_*E*_ = 0.12%. These values summarize the contribution from the two types of connectors (Fig 2H). For long-range specific interactions (Fig 3), we previously obtained *β*_*D*_ = 1.02, *β*_*E*_ = 0.99, corresponding to *ξ*_*D*_ = 0.12%, *ξ*_*E*_ = 0.07%(Fig 2H) for TAD D and E, respectively. Therefore, we attributed the remaining percentages to the addition of random connectors, that is *ξ*_*D*_ = 0.11, *ξ*_*E*_ = 0.05. The number of added random connectors corresponding to *ξ*_*D*_ = 0.11%, *ξ*_*E*_ = 0.05% are 6 and 10 in TAD D and E, respectively.

To reconstruct the polymer, we started with a Rouse chain (Fig 4A (gray)) and added 24 connectors between monomer pairs corresponding to peaks of the EP matrix (red). Three polymer realizations, simulated with the two types of connectors, are shown in Fig 4B, characterized by a radius of Gyration *R*_*g*_ = 6.4. We computed the EF-matrix (Fig 4C) that showed similarity with the experimental data (compare Fig 1A with Fig 4B), for which two TAD-like structures are visible. We find a satisfactory agreement between simulations and experimental data, measured by the Kolmogorov-Smirnov distance *D*_*Max*_ = 0.06 computed on the cumulative density function between the reconstructed and experimental data, as shown in S1 Fig).

We further quantified the similarity between the two matrices by comparing the decay exponent for each monomer *β*_*n*_, (*n* = 1‥307) from numerical simulations to those of the experimental data. We used the function 2 to fit the EP of monomers 1–307 after long time polymer simulations (Materials and Methods). The fitted value for *β* shows an good agreement with the experimental *β* values (Fig 3C). Comparing the normalized difference between *β* signals we find ‖*β*_*Exp*_ − *β*_*Sim*_‖_1_/‖*β*_*Exp*_‖_1_ = 0.2 (compared with 0.312 Fig 3B). To conclude, accounting for long-range deterministic and short-range stochastic interactions leads to a more accurate polymer model for chromatin reconstruction. The quality of this approximation is measured by the decay norm of the *β* exponent between the data and the simulations, also confirmed by the Kolmogorov-Smirnov distance as shown in S1C Fig and to be compared with S1D Fig.

### Encounter probabilities and distribution of search times of three genomic sites

**Fig 5:**
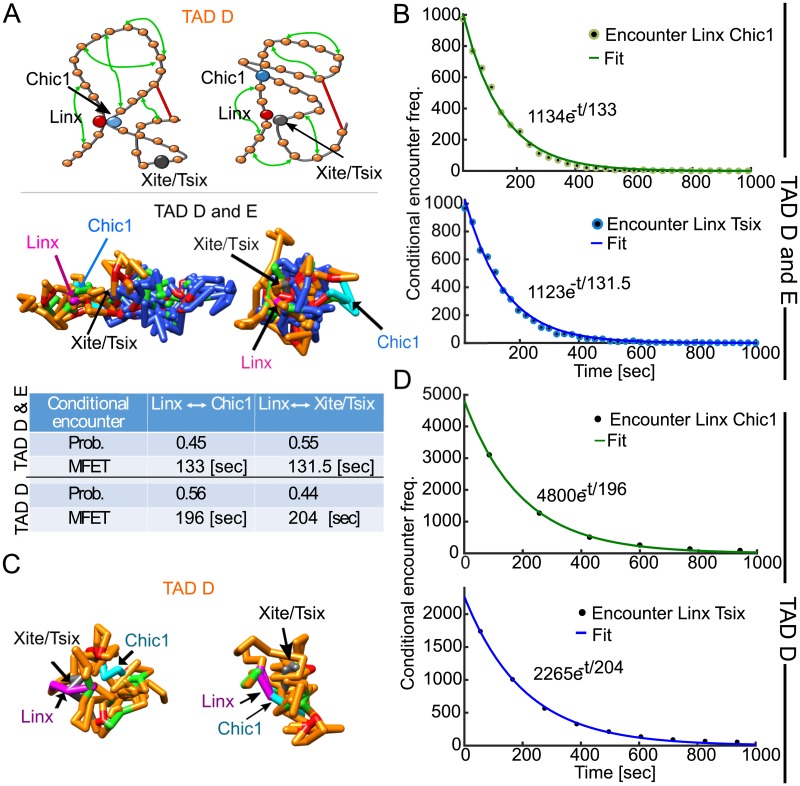
Transient properties of the chromatin: Conditional mean time and probability for three sites to meet. **A**. (upper panel) Representation of the polymer model for TAD D (orange, monomers 1–106), where loci Linx (monomer 26, red) meets Chic1 (monomer 62, cyan) and Xite/Tsix (monomer 87, gray), respectively. Random connectors (green arrows) and specific long range-connectors (red bar) are added, following the connectivity *ξ*. Fixed connectors (red bars) correspond to specific peaks of the 5C data. Two realizations (bottom panel) of the polymer model containing TAD D and E, show the encounter of Linx (magenta) with Chic1 (cyan), and Xite/Tsix (gray), respectively. The color code is from the upper panel. **B**. Histogram of the conditional encounter times between Linx and Chic1 (upper panel, green), and Linx and Xite/Tsix (bottom panel, blue) with TAD D and E. **C**. Two polymer realizations with a single TAD D (monomers 1–106, orange), showing the encounter between Linx (magenta) and Xite/Tsix (gray, left panel), and the encounter between Linx and Chic1 (cyan, right panel). **D**. Histogram of the conditional encounter times for a polymer with only TAD D, showing an exponential decay as in sub-figure B.

We showed previously how to construct a polymer, the statistical properties of which match the ones extracted from 5C data. However, the 5C data cannot be used to study transient properties of the chromatin, as they represent a static genomic encounter interactions averaged over cell population. We shall now use the calibrated polymer described above, constrained by the steady-state properties of the 5C data (Fig 5A), to evaluate transient properties of the chromatin.

We focus here on the 5C data harboring the Xist loci, which is the master regulator of X chromosome inactivation (XCI), and its antisense transcript, Tsix, which plays a key role in modulating Xist expression during mouse development [3, 5]. Tsix is believed to play a key role in the choice of the Xist allele that will be expressed during X inactivation. Thus, we decide to estimate how chromatin conformation within TADs can contribute to this transcriptional variability using the present polymer model. We estimated the first encounter time distribution and the probability that monomer 26 (position of the Linx) meets monomer 87 (Xite) before monomer 62 (Chic1). These monomers represent three key sites on the X chromosome [3, 5], located in TAD D. We show three realizations in Fig 5A and indicate the location of the three sites inside TAD D (yellow) and E (blue).

We started the polymer simulations from the steady-state distribution and performed around 10,000 runs. As predicted by the narrow escape theory [31, 32], the encounter time between two of the three monomers is Poissonian, and we confirmed this result by simulating the distributions (Fig 5B). The reciprocal of the mean encounter time is by definition [33] the encounter rate that we extract in Fig 5B–5D. We found also that the encounter probability, computed from the encounter rates (rate divided by the sum of the rates) as between Linx and Chic1 is *P* = 0.55, while the mean encounter times between each pair (Linx-Chic1) and (Xist-Linx) are comparable of the order of 131s (table C in Fig 5).

Finally, to check the impact of TAD E on the encounter time inside TAD D, we ran another set of stochastic simulations after removing TAD E (Fig 5D). Surprisingly, the encounter probability was inverted compared to the case of no deletion, while the mean time was increased by almost 50% to 195s (Linx to Chic1) and 205s (Linx to Xite). This result suggests that specific long-range interactions between TAD D and E (table in S1 Text) serve to modulate the probability and the encounter time between the three key genomic sites. This result further indicates that the search time inside a TAD can be influenced by neighboring chromatin configurations.

### Statistics of single loci trajectories in the reconstructed polymer model

**Fig 6:**
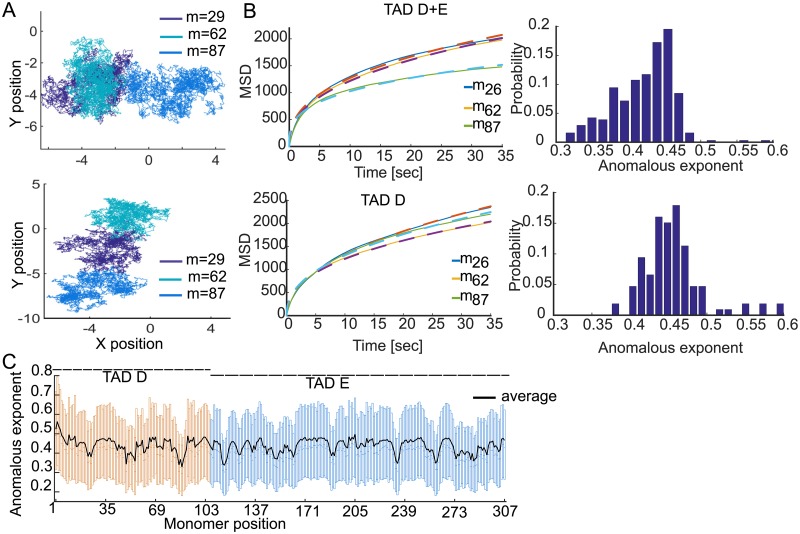
Statistics of SPTs simulated from the reconstructed polymer model. **A**. Representation of the polymer model described in Fig 5 for TAD D (orange, monomers 1–106). Trajectories for the three loci Linx (monomer 26, red) Chic1 (monomer 62, cyan) and Xite/Tsix (monomer 87, gray). **B**. MSD of trajectories shown in **A** for TAD D + E (left, upper) and TAD D alone (left, lower). The anomalous exponents for the three loci are *α*_26_ = 0.39, *α*_62_ = 0.4, and *α*_87_ = 0.31 (TAD D + E), while the anomalous exponent become $\alpha_{26}^{-E}=0.46$, $\alpha_{62}^{-E}=0.4$, $\alpha_{87}^{-E}=0.43$, when TAD E is removed. The histograms of the anomalous exponent (right column) is computed by averaging over 500 realizations with different random connectors positions. **C**. Box plot of the anomalous exponents (25–75%) computed over 500 realizations by changing the random connectors locations.

To further study the statistical properties of loci trajectories, we simulated the three loci monomer 26 (Linx), monomer 87 (Xite) and 62 (Chic1), following classical single particle tracking experiments (Fig 6A). We used the calibrated polymer model reconstructed in section Encounter probabilities and distribution of search times of three genomic sites. Starting from an equilibrium configuration following a relaxation time, we estimated the mean-square displacement (MSD) and computed the anomalous exponent over all realizations. The MSD of a stochastic process *X*(*t*) is computed by averaging over trajectory *X*_*i*_(*t*) realizations [33],

$$\begin{array}{c} {\left\langle |X\left(t\right)-X\left(0\right)\right|}^{2}\rangle =\lim_{N_{p}\to \infty }\frac{1}{N_{p}}\sum_{i=1}^{N_{p}}{\left|X_{i}\left(t\right)-X_{i}\left(0\right)\right|}^{2} \end{array}$$ (4)

and for short time, we use the asymptotic behavior

$$\begin{array}{c} {\left\langle |X\left(t\right)-X\left(0\right)\right|}^{2}\rangle ∼At^{\alpha }, \end{array}$$ (5)

where *A* is a constant and *α* is called the anomalous exponent. In practice, we computed the MSD from the estimator $\frac{1}{N_{p}}\sum_{i=1}^{N_{p}}{\left|X_{i}\left(t\right)-X_{i}\left(0\right)\right|}^{2}$, where the number of trajectories *N*_*p*_ is of the order 1000. We fitted the function *At*^*α*^ to the MSD Eq 5, computed on the simulated trajectories and then extracted *α*.

We explore now the consequence of computing the MSD when averaging over a cell population. To reproduce this situation, we consider a class of polymer configurations *C*_6,10_ obtained by fixing the number of random connectors 6, and 10 for TAD D and E respectively. However, their positions in each realization is allowed to vary, generating a variability. We shall now estimate the anomalous exponent *α*_*w*_ of a locus for a given configuration *w* ∈ *C*_6,10_ and then compute the average <*α*_*w*_>_*w* ∈ *C*_6,10__ over the ensemble *C*_6,10_. We shall emphasize that long-range specific connectors (table in S1 Text) are also accounted for in the polymer ensemble *C*_6,10_.

For two specific configurations from *C*_6,10_, one with TADs *E* + *D* (Fig 6A Upper) and the other TAD D alone (Fig 6A Lower), we show SPTs for each monomer *m* = 26, 62, 87, projected in two dimensions. We then computed the MSDs curves by averaging over all realization (Fig 6B). Further, the distribution of the anomalous exponents is shown in Fig 6C. Interestingly, we found that each locus *m* = 26, 62, 87 had a different mean anomalous exponent $\alpha_{26}^{D+E}=0.39$, $\alpha_{62}^{D+E}=0.4$, $\alpha_{87}^{D+E}=0.31$, revealing the intrinsic heterogeneity present in the chromatin. The *α* values we have computed for all monomers are indeed heterogeneous, as can be seen in histograms of Fig 6B (right column), 6C and S2A–S2B Fig left columns. Values are spreading in the range 0.3 to 0.5, however there is a peak in the histogram between values 0.4 to 0.45.

To evaluate how the anomalous exponent is influenced by persistent long-range interactions present in the EP data, we repeated polymer simulations and MSD analysis by removing TAD E. The distribution of the anomalous exponent *α* shows that removing TAD E results in the loss of low values, which characterize high chromatin connectivity (Fig 6B, right). We found the following changes in the anomalous exponents: $\alpha_{26}^{D}=0.46$, $\alpha_{62}^{D}=0.4$, $\alpha_{87}^{D}=0.43$. This result shows how specific long-range interactions affect the local chromatin dynamics and locus motion. Finally, the anomalous exponent averaged over all monomers is *α*^*D*+*E*^ = 0.425, whereas for TAD D and E we found *α*^*D*^ = 0.433, *α*^*E*^ = 0.42. However, when TAD E is removed, the average anomalous exponent for all monomers in TAD D is *α*^*D*^ = 0.458.

To represent the contribution of a single cell experiments in a population, we simulated SPTs for a fixed polymer configuration (we chose an ensemble of connectors in *C*_6,10_). We computed the MSD of all loci *n* = 1‥307 that can be divided into 3 classes: low medium and high anomalous exponent (SI). The distribution of the anomalous exponents of all sites is quite uniform (S2 Fig). We then vary the connector positions and computed the spread of the anomalous exponents (Fig 6C). This result shows that changing the connectors position account for the variability of the anomalous exponents. This result clarifies how the local chromatin organization affects the MSD when computed across cell population [21]. Indeed, random connectors model molecular binding that can vary from cell-to-cell.

To conclude, the construction of polymer models from 5C data can now be used to simulate SPTs of any loci of interest and thus to explore the inherent statistical variability found experimentally (Fig 6C). The present results can be used to interpret the variability of the MSD found in SPTs of live cell imaging, where the same locus in different cells can exhibit a different anomalous exponent, depending not only on the locus position, but also on the intrinsic variability due to the random arrangements of binding molecules between cells.

## Discussion

We presented here a general method to construct a coarse-grained polymer model from the 5C encounter probability (EP) matrix. This construction preserves the statistical properties of the 5C data, such as the decay rate of the EP of each monomer. The present approach is not used to study the configuration space of chromatin geometry, because it is too large to be fully sampled by elementary polymer models. However, we used this approach to generate statistics of passage times and radius of gyration, which characterize more accurately chromatin dynamics and are not contained in the 5C data. We built here a coarse-grained polymer model of the chromatin based on Rouse and we disregarded the repulsion forces between monomers and possible cross over of bonds that certainly influences the dynamics of the chromatin and statistical results. Future models should clearly examine the effect of repulsion forces on the present reconstruction, especially to study refined spatial scales below few kbp. We added connectors between random monomer-pairs to characterize sub-configurations present in 5C data. Connectors are represented by springs between monomer-pairs and account for the chromatin architectures. By adding connectors between random monomers, we were able to recover TAD sub-regions. By randomizing connectors positions, we could reproduce the inherent variability in chromatin architecture of nuclei population captured in 5C experiments.

Using our methodology, the characteristics of the reconstructed polymer are derived directly from the empirical data and do not require any minimization procedure [5]. One of the key result here is to determine the number of connectors directly from the experimental EP decay (Fig 2). Connectors can directly be interpreted as molecular interactions mediated by proteins such as cohesin, condensing and CTCF bindings. For example, cohesin could bind at random places scattered along the chromatin at 5C data acquisition time. These bounds could generate TADs, as shown here using simulations (Fig 2). 5C Contact maps represent steady-state distribution obtained from looping events in large ensemble of millions of cells, where TAD structures appears. The present approach differs from classical reconstruction methods, where 3D structures of a genome are inferred from 5C contact frequency data [34–37]. Previous models explored the effect of connectors between regions of the chromatin [18, 38] and examined the consequences on the EP-decay rate, but the positions and the number of these connectors were not derived from data. Here, we use connectors to resolve a reverse engineering problem, which is to recover the degree of connectivity from the EP-decay rate (Fig 2). The relation between the mean number of connectors and the decay exponent *β* of the EP (Eq 2) is found using simulations in section Encounter probability in the random loop polymer model. The decay exponent of the EP characterizes the polymer scaling statistics [38] and for this reason, the present model extends the key switch-and-binders model developed in [38].

It was surprising to find that a TAD subregion could influence the encounter distribution between monomers located in different TADs (Fig 5). Indeed, the distribution of looping time in free space depends only on the distance between monomers, while in confined domain, the nuclear boundary has an effect [30, 32, 39]. We found here that this modulation of loop regulation is due to long-range inter-TAD interactions, that are present in the 5C data and accounted for here in the construction of the polymer model. Indeed, we reported (SI) significant inter-TAD interactions between three monomer pairs: 86–234, 86–260, 24–285, where spring constants, representing long-range interactions are at least twice larger than other interactions. The threshold procedure described in Material and Methods (Eq 11) disregards the peaks of the 5C data for which the EP falls below the nearest-neighbor threshold *T*_*th*_. The peaks in the 5C below *T*_*th*_ were ignored because we interpret them as transient events, or a statistic that was not shared by the majority of the chromatin events or it could simply be due to some random fluctuations in the data. Long-range stable interactions have also been found in other mammalian chromosomes [4].

Finally, the present polymer reconstruction allows probing the dynamics of single particle trajectories (SPTs). We use our reconstructed polymer model to explore transient properties and the encounter time distribution between any two sites. Additionally, we simulated single particle trajectories from a reconstructed polymer model (Fig 4) and estimated the anomalous exponents, following the routine procedure in experimental SPTs. The present analysis reveals that the variability of the anomalous exponent of a given loci is due to the heterogeneity of the local polymer configurations constructed from the 5C data. This heterogeneity is simulated here as the random cross-links between monomer pairs. Indeed, for a Rouse polymer or any uniformly connected polymers, the anomalous exponent is constant [30, 40, 41].

We suggest that the inherent fluctuations of the chromatin interactions can be due to the random positions of binding molecules. Indeed, even for a fixed number of connectors, there still remains an intrinsic variability of monomer binding positions, leading to the same EP decay rate. This structural heterogeneity originating from connector positions certainly influences the anomalous exponents and can be used to re-interpret experimental SPTs [19, 21, 42, 43]. The present method and algorithms are generic and can be used to reconstruct a polymer model at a given scale (number of monomers and number of bps coarse-grained in a monomer) in a limit of few Mbp. Analyzing chromatin condensation and its transient properties can now integrate chromosomal capture data and SPTs statistics.

## Materials and methods

### Presentation of a generalized Rouse polymer model with long and short-range connections

The Rouse model [10] describes a polymer as a collection of beads *R*_*n*_(*n* = 1…*N*) linearly connected by harmonic springs and driven by Brownian motion. The energy of the polymer is given by [10]

$$\begin{array}{c} \phi_{Rouse}\left(R_{1},‥,R_{N}\right)=\frac{1}{2}\sum_{j=1}^{N-1}\kappa {\left(R_{j}-R_{j+1}\right)}^{2}, \end{array}$$ (6)

where $\kappa =\frac{3k_{B}T}{\gamma b^{2}}$ and *b* is the standard deviation of the distance between adjacent monomers, *γ* is the friction coefficient, *k*_*B*_ the Boltzmann coefficient, and *T* the temperature.

To account for a sub-chromatin region $C_{N}$, characterized by a higher EP than the rest, we will add connectors between monomer-pairs chosen randomly (with uniform distribution) inside this subregion such that an additional potential

$$\begin{array}{c} \phi_{Rand}\left(R_{1},‥,R_{N}\right)=\frac{1}{2}\sum_{j,k\in C_{N}}\kappa {\left(R_{j}-R_{k}\right)}^{2}, \end{array}$$ (7)

is added to *ϕ*_*Rouse*_, where $C_{N}$ is the ensemble of indices defining the sub-region. The number of connectors is a free parameter, that will be determined from experimental 5C data.

In addition, to account for consistent long-range interactions, reflected by peaks in EP matrix (Fig 1C), we will fix connectors between monomer-pairs by adding a spring constant *κ*_*m*, *n*_ between monomer *m* and *n*, so that the associated energy related to the peak interactions is described by

$$\begin{array}{c} \phi_{Peaks}\left(R_{1},\ldots ,R_{N}\right)=\frac{1}{2}\sum_{n,m\in S_{Max}}\kappa_{n,m}{\left(R_{n}-R_{m}\right)}^{2}. \end{array}$$ (8)

We will discuss below how the spring constant *κ*_*m*, *n*_ is computed from empirical data. In summary, the total energy of a polymer containing random connectors and prescribed peaks, is the sum of three energies Eqs 6, 7 and 8:

$$\begin{array}{c} \Phi \left(R_{1},‥,R_{N}\right)=\phi_{Rand}\left(R_{1},‥,R_{N}\right)+\phi_{Peaks}\left(R_{1},\ldots ,R_{N}\right)+\phi_{Rouse}\left(R_{1},‥,R_{N}\right) \end{array}$$ (9)

and the stochastic equation of motion for *n* = 1, ‥, *N* is

$$\begin{array}{c} \frac{dR_{n}}{dt}=-\nabla_{R_{n}}\Phi \left(R_{1},‥,R_{N}\right)+\sqrt{2D}\frac{d\omega_{n}}{dt}, \end{array}$$ (10)

where $D=\frac{k_{B}T}{\gamma }$ is the diffusion constant, *γ* is the friction coefficient, and *ω*_*n*_ are independent 3-dimensional Gaussian noise with mean 0 and standard deviation 1.

### Polymer parameter calibration from 5C data

To account for the 5C-data, comprised of a subsection of the X-chromosome from female mice embryonic stem cells reported in [3], showing TAD D and E as two diagonal blocks (Fig 1A), we use the coarse-graining procedure of [5], with a polymer of length *N* = 307. Each monomer represents a genomic segment of 3*kbp* and is connected to its 2 nearest neighbors by a harmonic spring (see subsection above). TAD D (resp. E) is represented by the range of monomers from 1 to *N*_*D*_ = 106 (resp. 107 to 307), and *N*_*E*_ = 201 for TAD E.

To reproduce the empirical EP extracted from data (see formula 1), we connected non-nearest neighbor pairs of monomers chosen randomly with uniform probability $\frac{1}{N_{1}}$ (resp. $\frac{1}{N_{2}}$)) where *N*_1_ = (*N*_*D*_ − 2) (*N*_*D*_ − 1)/2 (resp. *N*_2_ = (*N*_*E*_ − 2) (*N*_*E*_ − 1)/2). The number of connectors in each TAD is a percentage *ξ* of the total possible number of non nearest neighbor pairs, for TAD D (resp. E), we have $C_{D}=\xi_{D}\frac{N_{1}}{100}$ (resp. E $C_{E}=\xi_{E}\frac{N_{2}}{100}$) and *ξ*_*D*_, *ξ*_*E*_ ∈ [0, 100], which will be extracted from data. Random connectors were not added between monomers belonging to different TADs.

The procedure of adding random loops to a Rouse polymer is implemented using the energy of random loops, as described in the previous subsection. Finally, 24 connectors were added (SI) in all polymers, corresponding to the selected peaks present in the EP matrix, obtained in the following procedure: we located the positions of peaks that form a subset *S*_*Max*_ of the ensemble of the off-diagonal local maxima in the EP-matrix, such that their EP is higher than a threshold *T*_*th*_. In practice, we assumed that the encounter probability between neighboring monomers is almost not affected by the global chromatin structure. Therefore, any EP value in the matrix *M*_*i*, *j*_ above the threshold *T*_*th*_ (equals to the the EP of the nearest neighbor monomers) is considered to be a stable loop. The threshold *T*_*th*_ is defined as follows:

$$\begin{array}{c} T_{th}=\frac{\sum_{i}M_{ii-1}+M_{ii+1}}{\sum_{i,j}M_{ij}}. \end{array}$$ (11)

In a second step, we determine the spring constants *κ*_*m*, *n*_ between monomer *m* and *n* in the ensemble *S*_*Max*_ from the empirical EP *P*_*m*, *n*_ at each peak position. We recall that for a Rouse chain the joint probability density function of beads *R*_*m*_ and *R*_*n*_ is given by [10, p.15]

$$\begin{array}{c} \Phi \left(R_{m},R_{n},\Delta_{m,n}\right)={\left(\frac{3}{2\pi b^{2}\Delta_{m,n}}\right)}^{3/2}\exp\left(-\frac{3{\left(R_{m}-R_{n}\right)}^{2})}{2b^{2}\Delta_{m,n}}\right) \end{array}$$ (12)

where Δ_*m*, *n*_ = |*m* − *n*|. We assume that the encounter probability between neighboring monomers is not affected by global polymer structure, thus for the nearest neighbors Δ_*m*, *n*_ = 1 the EP occurs at small distances such that the exponential term is almost 1, that is

$$\begin{array}{c} P_{m,n}={\left(\frac{3}{2\pi b^{2}}\right)}^{3/2}\approx {\left(\frac{\kappa_{m,m+1}}{2\pi }\right)}^{3/2}, \end{array}$$ (13)

We approximate the chromatin structure as a polymer chain with a variance *b*^2^ between adjacent monomers. Thus, the constant $\bar{b}$ is estimated as the mean EP over the sub- and super-diagonals:

$$\begin{array}{c} {\left(\frac{3}{2\pi {\bar{b}}^{2}}\right)}^{3/2}\approx \sum_{i}\left(P_{ii-1}+P_{ii+1}\right)=\frac{\sum_{i}M_{ii-1}+M_{ii+1}}{\sum_{i,j}M_{ij}}=T_{th} \end{array}$$ (14)

To account for long-range interactions, we applied formula 13 to estimate the effective spring constant from the empirical EP $\tilde{P}$, so that

$$\begin{array}{c} \kappa_{m,n}=2\pi {\tilde{P}}_{m,n}^{2/3}. \end{array}$$ (15)

Finally, the energy related to peak interactions is described by

$$\begin{array}{c} \phi_{Peaks}\left(R_{1},\ldots ,R_{N}\right)=\frac{1}{2}\sum_{n,m\in S_{Max}}\kappa_{n,m}{\left(R_{n}-R_{m}\right)}^{2}. \end{array}$$ (16)

### Numerical simulations of the reconstructed polymer model

Using the reconstruction method described in the two previous subsections, we generate polymer realizations, each differs in the position of random connectors inside a TAD. To generate statistics from the EP matrix, we started from an initial random walk configuration and simulated the polymer until its equilibrium after a relaxation time *τ*_*R*_. The time *τ*_*R*_ is determined for each realization from the formula *τ*_*R*_ = 1/(*κ*_*min*_*λ*_1_), where *κ*_*min*_ is the minimal positive spring constant, and *λ*_1_ is the smallest non-vanishing eigenvalue of the polymer’s connectivity matrix [44], which we computed numerically (in practice it is of the order of thousands of simulation steps with Δ*t* = 0.01*s*).

For the numerical simulations, we divide Eq 10 by $\sqrt{D}$ and the spring constants are scaled by the friction coefficient such that $\kappa =\frac{dk_{B}T}{\gamma b^{2}}=\frac{dD}{b^{2}}$, in dimension *d* = 3. The encounter frequency matrix of the 307 monomers is computed at time *τ*_*R*_, where two monomers are considered to have encountered if their distance is less than *ϵ*. The time step for all simulations is Δ*t* = 10^−2^ *s*. The value of the parameter *b* is computed using formula 14. Using the *b* value in the relation *κ* = 3*k*_*B*_*T*/*b*^2^ [10], we computed the spring constant for random connectors and the linear backbone of the polymer. The spring constants for persistent long-range connectors were estimated using Eq 15 and are summarized in the table in S1 Text, other simulation parameters are summarized in Table 1.

**Table 1:** Values of simulation parameters.

Parameter	Value	Description
d	3	Dimension
b	0.6 [*μm*]	STD of distance between adjacent monomers
D	4 × 10^−2^ [*μm*^2^/*s*]	Monomer diffusion coefficient [45]
*ϵ*	0.03 [*μm*]	Encounter distance
*γ*	3.1 × 10^−5^ *Ns*/*m*	Friction coefficient [45]
*κ*	3 × 10^−5^ *N*/*m*	Spring constant
Δ*t*	10^−2^ *s*	Time step

## Supporting information

S1 Text Supplementary information. This supplementary information contains three sections. In the first section, we summarize in a table the spring constants associated to long-range connections used in Figs 3–6 of the main text. In the second section, we present the procedure for comparing the experimental encounter probability (EP) matrix with simulations. In the third section, we discuss the distribution of anomalous exponents and the Mean-Square-Displacement (MSD) computed over the simulated single particle trajectories (SPTs). These trajectories are generated by the polymer model reconstructed from the 5C data. This part links chromosomal capture data to SPTs.
(PDF)

S1 Fig Simulations and experimental 5C encounter matrices. **A**. Three-dimensional representation of the empirical encounter probability matrix **B**. Three-dimensional representation of the simulation encounter probability matrix for polymer with persistent long-range and random connectors. **C**. Cumulative distribution function of monomer encounters for a polymer model with only persistent long-range connectors, simulations (blue) vs. experimental (orange) data. The Kolomogorov-Smirnov distance is *D*_*max*_ = 0.15. **D**. Cumulative distribution function of monomer encounter for a polymer model with persistent long-range and random connectors, simulations (blue) vs. experimental (orange) data. The Kolmogorov-Smirnov distance is *D*_*max*_ = 0.06.
(EPS)

S2 Fig MSD and anomalous exponent of polymer realizations. **A**. Distribution of the anomalous exponent for 3 polymer realizations (left column). The MSD curves for each realization is shown in the right column. MSD curves are classified into 3 classes: low (blue), medium (green) and high (red) based on the MSD value at time 35 sec. **B**. Distribution of anomalous exponent (right column) for TAD D, when TAD E is removed. The distribution in each class is given by low (blue, 85%) medium (green, 12% of curves) and high (red, 1% of curves).
(EPS)
