# Supplementary material for: Transient chromatin properties revealed by polymer models and stochastic simulations constructed from Chromosomal Capture data
Source: PLoS Comput Biol. 2017 Apr 3;13(4):e1005469. doi: 10.1371/journal.pcbi.1005469 (PMC5393903; doi:10.1371/journal.pcbi.1005469)
Supplement: S1 Text — This supplementary information contains three sections. In the first section, we summarize in a table the spring constants associated to long-range connections used in Figs 3–6 of the main text. In the second section, we present the procedure for comparing the experimental encounter probability (EP) matrix with simulations. In the third section, we discuss the distribution of anomalous exponents and the Mean-Square-Displacement (MSD) computed over the simulated single particle trajectories (SPTs). These trajectories are generated by the polymer model reconstructed from the 5C data. This part links chromosomal capture data to SPTs. (PDF) [file pcbi.1005469.s001.pdf]

Supplementary Information:
Transient chromatin properties revealed by polymer models and stochastic simulations constructed from Chromosomal Capture data

Ofir Shukron[1], David Holcman[1,2*]

1 Institute of Biology, Ecole Normale Supérieure, Paris, France
2 Mathematical Institute, University of Oxford, Oxford, United Kingdom

* david.holcman@ens.fr

This supplementary information contains three sections. In the first section, we summarize in a table the spring constants associated to long-range connections used in Fig 3-6 of the main text. In the second section, we present the procedure for comparing the experimental encounter probability (EP) matrix with simulations. In the third section, we discuss the distribution of anomalous exponents and the Mean-Square-Displacement (MSD) computed over the simulated single particle trajectories (SPTs). These trajectories are generated by the polymer model reconstructed from the 5C data. This part links chromosomal capture data to SPTs.

Values for the spring constants of long-range monomer interaction

Table 1 summarizes the values of the spring constants $\kappa_{m,n}$ that we computed from the EP matrix (see Materials and methods) for simulating long-range connectors between monomer m and n. The right column indicates which TADs are connected by the monomer-pairs. The values are 1.1 to 3 times higher than the spring constant of randomly added connectors, and for adjacent monomers in the linear backbone (nearest-neighbor connection) of the polymer ($\kappa = 3 \times 10^{-5} Nm^{-1}$).

Comparison of the experimental and simulation encounter data

We now compare the EP matrices about the steady-state of our models and the experimental data. We compare the experimental EP matrix (S1A Fig) with the simulation of the model with persistent and random connectors (S1 Fig B). The simulated EP matrix is much smoother than the experimental 5C EP matrix.

To estimate the similarity between the EP matrices, we computed the cumulative distribution function (CDF) of the monomer encounters. The CDF is computed by averaging the EP between all monomers m and n from an encounter frequency matrix M. For each row of M, we start from the diagonal entry and average counts at symmetrical positions. The EP is given by

$$P(|m-n||n) = \frac{M_{n,n+|m-n|} + M_{n,n-|m-n|}}{\sum_{m=1}^{N} M_{n,m}}, \tag{1}$$

Spring const.	value$\times 10^{-5}[Nm^{-1}]$	connecting TADs
$\kappa_{14,10}$	3.9555	$D \leftrightarrow D$
$\kappa_{48,44}$	4.1396	$D \leftrightarrow D$
$\kappa_{54,50}$	3.6777	$D \leftrightarrow D$
$\kappa_{60,56}$	5.9841	$D \leftrightarrow D$
$\kappa_{69,65}$	3.1722	$D \leftrightarrow D$
$\kappa_{79,75}$	3.0864	$D \leftrightarrow D$
$\kappa_{95,91}$	4.6673	$D \leftrightarrow D$
$\kappa_{113,109}$	3.2280	$E \leftrightarrow E$
$\kappa_{126,117}$	3.4386	$E \leftrightarrow E$
$\kappa_{144,136}$	3.6859	$E \leftrightarrow E$
$\kappa_{151,50}$	3.2399	$E \leftrightarrow D$
$\kappa_{161,157}$	3.4420	$E \leftrightarrow E$
$\kappa_{190,162}$	8.3386	$E \leftrightarrow E$
$\kappa_{205,201}$	3.5671	$E \leftrightarrow E$
$\kappa_{217,213}$	3.1621	$E \leftrightarrow E$
$\kappa_{234,86}$	4.2015	$D \leftrightarrow E$
$\kappa_{259,255}$	3.4765	$E \leftrightarrow E$
$\kappa_{260,86}$	7.2143	$D \leftrightarrow E$
$\kappa_{275,116}$	3.5335	$E \leftrightarrow E$
$\kappa_{279,275}$	3.2174	$E \leftrightarrow E$
$\kappa_{285,24}$	8.4070	$D \leftrightarrow E$
$\kappa_{293,289}$	4.7508	$E \leftrightarrow E$
$\kappa_{302,294}$	7.1703	$E \leftrightarrow E$
$\kappa_{305,301}$	3.1879	$E \leftrightarrow E$

Table 1. Spring constants for long-range interactions. Long-range fixed connectors added between monomers pairs indices (left column) and their computed spring constant (middle column, see Methods in main text), form connections within and between TADs as indicated in the right column

We average over $N = 307$ rows of the matrix, leading the CDF defined by

$$F(k) = \frac{1}{N} \sum_{m=1}^{k} \sum_{n=1}^{N} P(|m-n|n). \tag{2}$$

We use Eq 2) to compare the EP matrices, by computing the CDF for the experimental $F(k)_{Exp}$ and the simulation $F(k)_{Sim}$ data respectively. Finally, we shall use the Kolmogorov-Smirnov (KS) distance, defined by

$$D_{max} = \max_{k} |F(k)_{Exp} - F(k)_{Sim}|. \tag{3}$$

The KS distance is computed for the simulation of the model with persistent long-range connectors (subsection 3.3 of the main text) that we compare with the model with persistent long-range and random connectors (subsection 3.4 of the main text). In S1 Fig C, we show the CDF of the model with only persistent connectors gives $D_{max} = 0.15$. The CDF of the model that contain both persistent and random connectors (S1 Fig D), leads to an improved agreement with experimental data indicated by the value $D_{max} = 0.06$ at a level of 0.001 (P-Value= 0.06).

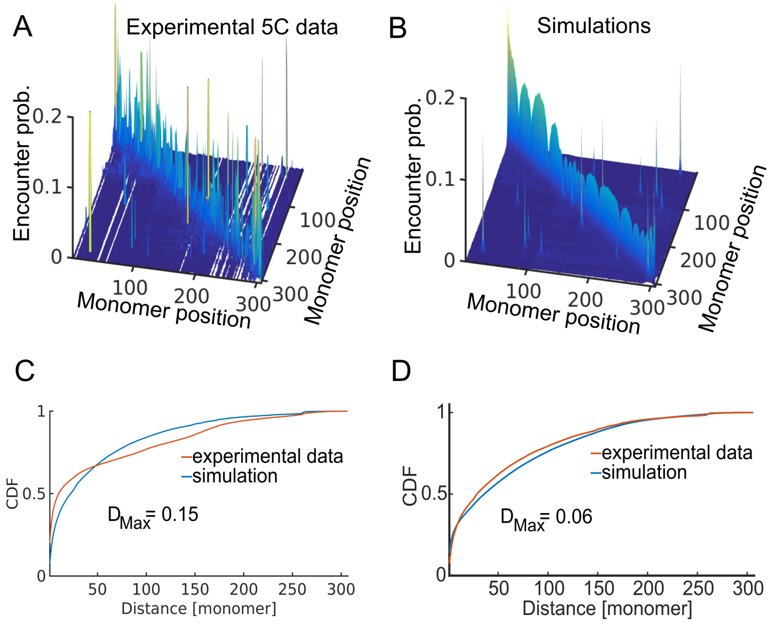

Fig 1. Simulations and experimental 5C encounter matrices. **A**. Three-dimensional representation of the empirical encounter probability matrix **B**. Three-dimensional representation of the simulation encounter probability matrix for polymer with persistent long-range and random connectors. **C**. Cumulative distribution function of monomer encounters for a polymer model with only persistent long-range connectors, simulations (blue) vs. experimental (orange) data. The Kolomogorov-Smirnov distance is $D_{max} = 0.15$. **D.** Cumulative distribution function of monomer encounter for a polymer model with persistent long-range and random connectors, simulations (blue) vs. experimental (orange) data. The Kolmogorov-Smirnov distance is $D_{max} = 0.06$.

MSD and anomalous exponent statistics for single polymer realization

We describe in this section the MSD along single monomer trajectories for polymer realizations having connectivity matching the one calibrated from the 5C data based on 6 and 10 randomly connectors for TAD D and E, respectively (this construction is already described in Fig 4 of the main text). Long-range connectors are included, as listed in Table 1. The anomalous exponent values for each single monomer trajectory is estimated by fitting a power law to the MSD:

$$f(t) = At^{\alpha}. \tag{4}$$

To each MSD curve, we computed the two parameters A and α (anomalous exponent). The values of the anomalous exponents are distributed in [0,1]. However, if we average over realizations of random connector positions, the anomalous exponents are concentrated around the mean 0.42 (Fig 6 of the main text), as shown for three realization in S2 Fig.

We classify the curves into low, medium and high MSD trajectories, by dividing equally the range of values at time $35s$ into 3 regions (S2A Fig right column) for each realization. The majority of the MSD curves (92.3%) saturate for long-time simulations (blue in S2A Fig right column), while a fraction 7.3% had a mix saturated an linear behavior. Finally, only a small fraction of 0.5% curves (red) are characterized by a rapid increase. S2A Fig right column shows an example of the MSD curves with three realizations.

We repeated the MSD analysis and the computation of the anomalous exponent when TAD E was removed. We found that the values of the anomalous exponent remained distributed in the range [0,1] (S2B Fig left column), similar to the case where TAD E is included. The MSD curves were divided into 3 classes as described above (S2B Fig right column), and we found that the majority of them (87%) saturate (blue), the medium class included 12% of the curves (green), which displayed mixed saturated and rapid MSD increase. Only 1% of the MSD curves were classified into high class (red curves), displaying rapid MSD increase, similar to the case with TAD E included.

We conclude that the distribution of anomalous exponents extracted from SPTs depends on the polymer configuration generated by random connectors. In addition, the distribution of MSDs in TAD D was sensitive to the influence of TAD E (as shown in comparing S2A Fig with B). The exact scaling law that connect the anomalous exponent measured in SPTs with the EP decay exponent of the Chromosomal Capture data remains unknown, although it is now clear that increasing the chromatin connectivity, leading to a higher decay exponent is characterized by a smaller anomalous exponent.

Computational tools

The data analysis and stochastic simulations were performed using our codes on Matlab 2015. The source codes and description are now available on our website http://bionewmetrics.org/. For the chromatin visualizations in Figs 2-5 of the main text, we use the UCSF Chimera software version 1.11.

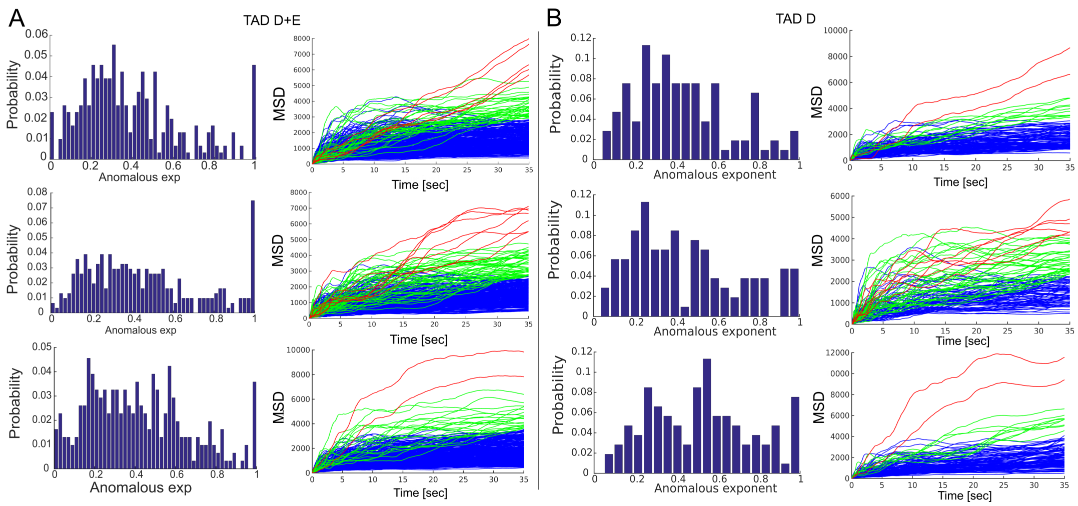

Fig 2. MSD and anomalous exponent of polymer realizations. **A.** Distribution of the anomalous exponent for 3 polymer realizations (left column). The MSD curves for each realization is shown in the right column. MSD curves are classified into 3 classes: low (blue), medium (green) and high (red) based on the MSD value at time 35 sec. **B** Distribution of anomalous exponent (right column) for TAD D, when TAD E is removed. The distribution in each class is given by low (blue, 85%) medium (green, 12% of curves) and high (red, 1% of curves).
